# Association between dietary acid load and metabolic health status in overweight and obese adolescents

**DOI:** 10.1038/s41598-022-15018-8

**Published:** 2022-06-24

**Authors:** Mahsa Rezazadegan, Saeideh Mirzaei, Ali Asadi, Masoumeh Akhlaghi, Parvane Saneei

**Affiliations:** 1 Department of Clinical Nutrition, School of Nutrition and Food Science, Food Security Research Center, Isfahan University of Medical Sciences, Isfahan, Iran; 2 Department of Community Nutrition, School of Nutrition and Food Science, Shiraz University of Medical Sciences, Shiraz, Iran; 3 Department of Exercise Physiology, School of Physical Education and Sport Sciences, University of Tehran, Tehran, Iran; 4 Department of Community Nutrition, School of Nutrition and Food Sciences, Shiraz University of Medical Sciences, Shiraz, Iran; 5 Department of Community Nutrition, School of Nutrition and Food Science, Food Security Research Center, Isfahan University of Medical Sciences, PO Box 81745-151, Isfahan, Iran

**Keywords:** Endocrine system and metabolic diseases, Nutrition disorders

## Abstract

The relationship between dietary acid load (DAL) and metabolic health status in adolescents has not been studied yet. We aimed to examine the association between DAL and metabolic health status in Iranian overweight/obese adolescents. This cross-sectional study included 203 overweight/obese adolescents selected by a multistage cluster random sampling method. Dietary intakes were assessed using a validated 147-item food frequency questionnaire (FFQ). Anthropometric indices and blood pressure values were measured. Fasting blood samples were obtained to determine glucose, insulin, and lipid profiles. Based on two methods (International Diabetes Federation (IDF) criteria and combination of IDF with Homeostasis Model Assessment Insulin Resistance (HOMA-IR)), participants were classified into metabolically healthy obese (MHO) or unhealthy obese (MUO). Adolescents in the highest tertile of potential renal acid load (PRAL) and net endogenous acid production (NEAP), compared with those in the lowest tertile, had 172% (95% CI 1.32–5.59) and 161% (95% CI 1.26–5.41) higher odds of MUO status, based on IDF criteria. This association was significant after adjustment for age, sex, and energy intake (PRAL: OR 2.42; 95% CI CI 1.13–5.15; NEAP: OR 2.52; 95% CI 1.17–5.41); but it disappeared after adjustment for other confounders. Based on IDF/HOMA-IR definition, there was a significant positive association between PRAL and being MUO only in the crude model (OR 2.37; 95% CI 1.13–4.96). The stratified analysis revealed that these associations for NEAP scores were stronger among overweight subjects than obese individuals, based on both metabolic status definitions. However, after adjustment for all potential confounders these relations were insignificant. Having higher DAL might be associated with higher odds of MUO phenotype in Iranian overweight/obese adolescents. More prospective studies are warranted to confirm this finding.

## Introduction

Overweight and obesity, defined as abnormal or excessive fat accumulation in the body, in childhood and adolescence has adverse health effects throughout the life^1^. In 2016, estimates showed that more than 330 million children and adolescents (aged 5–19 years) were overweight or obese^2^. Global burden of disease (GBD) data reported that by the year 2025, 268 and 124 million children and adolescents will be respectively overweight and obese^3^. According to a systematic review and meta-analysis published in 2020, 11% of Iranian students were generally obese (based on their body mass index (BMI))^4^. The existence of these conditions in early life is associated with psychosocial consequences, less educational attainment, and cardiometabolic disorders such as high blood pressure (BP), dyslipidemia and insulin resistance^5–7^. Also, childhood obesity can increase burden on health systems and reduce economic productivity later on^8^.

Recent attention has focused on two phenotypes for overweight and obesity including metabolically healthy overweight/obese (MHO) and metabolically unhealthy overweight/obese (MUO). As compared to their MUO counterparts, MHO subjects have better lipid profiles, insulin sensitivity, as well as lower cardiovascular disease (CVD) risk factors^9,10^. It would be possible to shift between metabolic health statuses^11^. Evaluation of metabolic health in the childhood period may be important to prevent adverse cardiometabolic diseases^12^. Physical activity, sleep, smoking and dietary intakes are factors that can affect metabolic health status^13^.

A previous study showed that the Dietary Approaches to Stop Hypertension (DASH) diet may improve insulin sensitivity in overweight children and adolescents^14^. Also, healthy eating patterns may have a positive effect on metabolically health status^15^. It is well-known that diet can have a significant effect on acid–base balance. When a diet is rich in animal products, cheese, cereals, and rice and low in fruits and vegetables, can result in endogenous acid production^16^. Acidosis has adverse health effects such as obesity, overweight, increased BP, and higher triglyceride (TG) levels^17,18^. Two scores are commonly used to measure dietary acid load (DAL) including the potential renal acid load (PRAL) score and net endogenous acid production (NEAP) score^19^. Women with higher NEAP scores had higher waist circumference (WC), serum TG, and weight in a cross-sectional study in Iran^20^. Furthermore, a study on adult Japanese workers found that higher PRAL and NEAP scores were associated with insulin resistance^21^. Another investigation conducted among young Japanese women demonstrated a link between a more acidic dietary load and the adverse profile of several cardiometabolic risk factors^22^. In case of children and adolescents, Aslani et al. illustrated a direct association between diet-induced acid load and neck circumference (NC) as well as an inverse association between DAL indices and parental BMI^23^. However, a recent study could not find a significant association between DAL and metabolic syndrome among children and adolescents^24^. Since there is no study on the association between DAL and metabolic health status in adolescents, the current study investigated the association of DAL with metabolic health status in Iranian overweight/obese adolescents.

## Methods

### Study design and population

This cross-sectional study included 102 girls and 101 boys (12–< 18 years old) who were selected by a multistage cluster random sampling method. Participants were randomly selected from 6 different districts of the city of Isfahan, Iran, using a stratified, multi-stage cluster sampling design. In total, 16 schools were chosen. Body weight and height of all students were measured and BMI was calculated based on weight (kg)/height^2^ (m). Then, adolescents were classified into 3 groups: normal-weight, overweight, and obese^25^. Overweight and obese students with different socioeconomic status (SES) were considered to participate in the study. The students who had the following conditions were not included in this study: having genetic or endocrine disorders (such as type 1 diabetes mellitus, hypothyroidism, and Cushing's syndrome), being on a weight-loss diet, taking vitamin and mineral supplements or medications that might affect body weight, hypertension, blood glucose or lipid profile. All participants and their parents signed the written informed consent. The study protocol was approved by the local Ethics Committee of Isfahan University of Medical Sciences.

### Assessment of dietary intakes

Dietary intake was assessed using a validated 147-item semi-quantitative food frequency questionnaire (FFQ)^26^. According to this questionnaire, dietary intake in the previous year was calculated by determining the frequency of consumption of each food item on a daily, weekly, or monthly basis. The amount of food consumed was evaluated by asking a standard common portion size. We converted the portion sizes of consumed foods to grams per day using household measures. Finally, the grams of food intakes were entered into Nutritionist IV software to derive nutrient intake data. Nutritionist IV software was based on USDA food composition data; some Iranian foods were also incorporated into it.

### Calculation of DAL indices

Urinary net acid excretion depends on dietary intake, and direct measurement is difficult. Therefore, two dietary indices (PRAL and NEAP) have been defined to calculate body acid production or DAL. In a previous study by Remer et al., the validity of both PRAL and NEAP scores compared to a 24-h urinary acid load was confirmed^27^. The PRAL score was calculated based on the following formula^27^:

$$ {\text{PRAL}}\,\left( {\text{mEq/day}} \right)\, = \,\left( {0.{4888}\, \times \,{\text{protein}}\,{\text{intake}}\,\left( {\text{g/day}} \right)} \right)\, + \,\left( {0.0{366}\, \times \,{\text{phosphorus}}\,\left( {\text{mg/day}} \right)} \right)\,{-}\,\left( {0.0{2}0{5}\, \times \,{\text{potassium}}\,\left( {\text{mg/day}} \right)} \right)\,{-}\,\left( {0.0{125}\, \times \,{\text{calcium}}\,\left( {\text{mg/day}} \right)} \right){-}\left( {0.0{263}\, \times \,{\text{magnesium}}\,\left( {\text{mg/day}} \right)} \right). $$

In addition, we estimated the NEAP score as follows^28^:

$$ {\text{NEAP}}\,\left( {\text{mEq/day}} \right) = \left[ {{54}.{5}\, \times \,{\text{protein}}\,{\text{intake}}\,\left( {\text{g/day}} \right)\, \div \,{\text{potassium}}\,{\text{intake}}\,\left( {\text{mEq/day}} \right)} \right]\, - \,{1}0.{2}. $$

### Assessment of anthropometric indices and cardiometabolic risk factors

Two trained nutritionists measured weight using a calibrated electronic scale (Seca Instruments, Germany) in minimal clothing and without shoes to the nearest 0.1 kg. Also, standing height was measured without shoes with a stadiometer to the nearest 0.1 cm. Then, body mass index was calculated as kg/m^2^. Based on the sex- and age-specific World Health Organization (WHO) cut-off points, participants were classified as overweight or obese adolescents^25^. Waist circumference was recorded twice at the midway between the lowest rib and the superior border of the iliac crest by the use of an unstretched flexible anthropometric tape at 0.1 cm precision, after a normal expiration and without any pressure on the body surface. The mean of the two measurements for each student was considered as WC. Systolic blood pressure (SBP) and diastolic blood pressure (DBP) were measured twice at the right arm using a mercury sphygmomanometer with a suitable cuff size after a rest period of 15 min. We considered the average of the two measurements in the analysis. Venous blood samples after 12 h of fasting were collected to determine biochemical values. Fasting blood glucose (FBG), high-density lipoprotein cholesterol (HDL-c), triglycerides (TG), and insulin concentrations were measured with standard methods. Homeostasis Model Assessment Insulin Resistance (HOMA-IR) was calculated to estimate Insulin resistance (IR) by using the following formula: HOMA-IR = (insulin (µUI/mL) × glucose (mg/dL))/405.

### Assessment of metabolic status

Two methods were used to classify participants into MHO or MUO individuals. Students with two or more of the four following risk factors were defined as MUO subjects, based on the modified International Diabetes Federation (IDF) criteria^29^: (1) increased fasting blood glucose (≥ 100 mg/dL), (2) increased blood pressure (≥ 130/85 mmHg), (3) increased triglycerides (≥ 150 mg/dL) and (4) decreased HDL-c (< 40 mg/dL for the age of < 16 y, and < 50 mg/dL in girls/ < 40 mg/dL in boys for the age of ≥ 16 y). MHO adolescents were those with one or no risk factors. In the second method, presence of insulin resistance (defined by HOMA-IR score) was added to the IDF criteria that were used in the first definition^30^. Students with HOMA-IR score ≥ 3.16 that had two or more above-mentioned metabolic risk factors were considered as MUO individuals and those with HOMA-IR < 3.16 were classified considered as MHO adolescents. Based on some previous studies on children and adolescents, the cut-off value for HOMA-IR was set at 3.16^31–33^.

### Assessment of other variables

The Physical Activity Questionnaire for Adolescents (PAQ-A), which contains 9 questions on various aspects of physical activity over the past week, was used to determine the physical activity level of participants^34^. Items 1 to 8 of this questionnaire were scored from 1 to 5, with 1 representing the least physical activity and 5 representing the most. The ninth question examined the unusual activity of adolescents during the previous 7 days. Based on their total scores, adolescents were categorized as highly active (score ≥ 4), moderately active (4 < score ≤ 3), lowly active (3 < score ≤ 2), sedentary, or not having an orderly week of physical activity (score < 2). A validated demographic questionnaire was used by trained investigators to ascertain SES of students (29), based on the following items: parental education level, parental job, number of family members, having cars in the family, having personal room, having computers/laptops and taking trips in the year. Finally, a total score of SES (ranged between 4 and 27) was calculated. Information of sex, age, history of diseases, and use of medications and dietary supplements of participants was gathered through a questionnaire.

### Statistical analysis

We presented continuous variables as mean ± SD/SE and qualitative variables as frequency (percentage). Participants were categorized into tertiles of PRAL and NEAP (based on their scores). We used one-way analysis of variance (ANOVA) and the χ2 test to compare quantitative and categorical variables between tertiles of PRAL and NEAP. Age-, sex- and energy-adjusted dietary macro and micronutrient intakes of participants were compared across tertiles of PRAL and NEAP using analysis of covariance (ANCOVA). Multivariable logistic regression was applied to identify the association between PRAL and NEAP with MUO status. The odds ratio (OR) and 95% confidence interval (CI) for MUO status were computed in crude and adjusted models. In the first model, we adjusted for sex, age, and energy intake. In the second model, further adjustments were done for physical activity levels and SES. In the last model, more adjustment for BMI was done. The first tertile of PRAL and NEAP was considered as the reference category, in all models. The overall trend of OR across increasing PRAL and NEAP tertiles was examined by considering tertiles of PRAL and NEAP as a continuous variable. Stratified analysis was done to obtain OR for MUO in different categories of BMI (overweight vs. obese individuals). SPSS software version 23 (IBM, Chicago, IL) was used for all analyses. *P* values < 0.05 (two-sided) were considered as statistically significant.

### Ethical approval and consent to participate

The study procedure was performed according to declaration of Helsinki and STROBE checklist. All participants provided informed written consent. The study protocol was approved by the local Ethics Committee of Isfahan University of Medical Sciences.

## Results

The present study included 101 (49.8%) boys and 102 (50.2%) girls with a mean age of 13.34 ± 1.38 and 14.61 ± 1.57 and BMI of 26.67 ± 2.82 and 28.03 ± 3.48, respectively. General characteristics and cardiometabolic risk factors of study participants across tertiles of PRAL and NEAP are presented in Table 1. Adolescents in the highest tertile of PRAL in comparison to those in the lowest tertile had higher FBG and TG (P < 0.05). Furthermore, subjects in the top category of PRAL had lower physical activity level and different SES status (P < 0.05). Adolescents in the highest tertile of NEAP had higher FBG than those in the lowest tertile and were different in terms of physical activity from those in the first category (P < 0.05). There were no significant differences in other general characteristics and cardiometabolic risk factors among tertiles of PRAL and NEAP.

**Table 1:** General characteristics and cardiometabolic factors of study participants across tertiles of PRAL and NEAP^1^.

	Tertiles of PRAL				Tertiles of NEAP			
	T1 (n = 67)	T2 (n = 68)	T3 (n = 68)	*P* value^2^	T1 (n = 67)	T2 (n = 68)	T3 (n = 68)	*P* value^2^
Range	< 6.21	6.21–17.46	> 17.46	–	< 48.52	48.52–62.35	> 62.35	–
**Sex, n (%)**								
Boys	32 (47.8)	31 (45.6)	38 (55.9)	0.44	37 (55.2)	33 (48.5)	31 (45.6)	0.51
Girls	35 (52.2)	37 (54.4)	30 (44.1)		30 (44.8)	35 (51.5)	37 (54.4)	
Age (year)	14.27 ± 1.73	13.68 ± 1.41	13.99 ± 1.62	0.10	13.97 ± 1.73	13.78 ± 1.53	14.18 ± 1.55	0.35
Weight (kg)	71.06 ± 9.61	74.19 ± 12.56	75.14 ± 12.15	0.10	71.23 ± 10.30	75.66 ± 12.54	73.50 ± 11.57	0.08
BMI (kg/m^2^)	26.82 ± 2.72	27.83 ± 3.95	27.39 ± 2.85	0.19	26.78 ± 2.86	28.04 ± 3.85	27.22 ± 2.80	0.07
Waist circumference (cm)	88.93 ± 7.31	90.82 ± 8.83	91.20 ± 7.49	0.20	89.13 ± 8.74	91.64 ± 7.84	90.19 ± 7.05	0.18
**Physical activity levels, n (%)**								
Sedentary	20 (29.9)	33 (48.5)	36 (52.9)	0.02	19 (28.4)	31 (45.6)	39 (57.4)	0.01
Low-activity	28 (41.8)	24 (35.3)	25 (36.8)		29 (43.3)	28 (41.2)	20 (29.4)	
Active	19 (28.3)	11 (16.2)	7 (10.3)		19 (28.3)	9 (13.2)	9 (13.2)	
**Socioeconomic status**^**3**^**, n (%)**								
Low	16 (23.9)	13 (19.1)	30 (44.1)	0.01	14 (20.9)	17 (25.0)	28 (41.2)	0.05
Medium	31 (46.3)	37 (54.4)	22 (32.4)		30 (44.8)	32 (47.1)	28 (41.2)	
High	20 (29.8)	18 (26.5)	16 (23.5)		23 (34.3)	19 (27.9)	12 (17.6)	
Systolic blood pressure (mmHg)	110.12 ± 24.98	113.18 ± 16.83	114.78 ± 10.27	0.32	110.48 ± 24.24	114.50 ± 18.07	113.10 ± 10.03	0.43
Diastolic blood pressure (mmHg)	71.80 ± 13.04	73.96 ± 10.84	74.69 ± 10.02	0.31	71.74 ± 13.14	75.41 ± 6.66	73.30 ± 12.99	0.17
Fasting blood glucose level (mg/dL)	95.16 ± 7.18	98.78 ± 7.81	100.41 ± 9.58	0.01	95.94 ± 7.57	98.25 ± 7.27	100.18 ± 9.98	0.01
Insulin (μUI/mL)	18.04 ± 12.12	20.78 ± 10.49	22.40 ± 14.77	0.12	18.88 ± 12.39	21.55 ± 14.43	20.81 ± 10.93	0.45
HOMA-IR index	4.34 ± 3.21	5.14 ± 2.92	5.55 ± 3.58	0.09	4.58 ± 3.32	5.26 ± 3.53	5.19 ± 2.96	0.41
Triglycerides (mg/dL)	108.78 ± 58.30	117.99 ± 53.61	138.90 ± 81.55	0.02	110.55 ± 60.89	123.97 ± 59.77	131.16 ± 76.87	0.19
HDL cholesterol (mg/dL)	45.51 ± 8.08	44.51 ± 7.36	44.46 ± 8.36	0.69	46.09 ± 7.93	43.88 ± 7.27	44.51 ± 8.47	0.25

^1^Values are Mean ± SD; unless indicated. Abbreviations: NEAP: net endogenous acid production; PRAL: potential renal acid load; BMI: Body Mass Index; HOMA-IR: Homeostasis Model Assessment Insulin Resistance; HDL-c: high-density lipoprotein cholesterol;

^2^*P* value for one-way analysis of variance (ANOVA) and χ2 test for quantitative and categorical variables, respectively.

^3^Socioeconomic status (SES) score was evaluated based on parental education level, parental job, number of family members, having car in the family, having computer/laptop, having personal room and having travel by using demographic questionnaire.

Dietary intakes of participants across tertiles of PRAL and NEAP are presented in Table 2. Participants in the top tertile of PRAL, compared to the bottom tertile, had greater intake of energy, protein, thiamin, niacin, selenium and sodium and lower intake of fat, MUFA, vitamin C, vitamin A, riboflavin, vitamin B_6_, folate, vitamin B_12_, magnesium, total fiber, potassium, and calcium. Also, highest vs. lowest tertile of NEAP was associated with increased intake of carbohydrate, thiamin and niacin. However, adolescents in the first tertile of NEAP, compared to those in the third tertile, had higher intake of fat, cholesterol, SFA, MUFA, vitamin C, vitamin A, riboflavin, vitamin B_6_, folate, vitamin B_12_, magnesium, zinc, total fiber, potassium, calcium and phosphorus.

**Table 2:** Dietary intakes (energy and macro/micro nutrients) of study participants across tertiles of PRAL and NEAP ^1^.

	Tertiles of PRAL				Tertiles of NEAP			
	T1 (n = 67)	T2 (n = 68)	T3 (n = 68)	P value^2^	T1 (n = 67)	T2 (n = 68)	T3 (n = 68)	P value^2^
Range	< 6.21	6.21–17.46	> 17.46	–	< 48.52	48.52–62.35	> 62.35	–
Energy, kcal	2756.28 ± 65.80	2894.76 ± 65.65	2996.16 ± 65.14	0.03	2826.52 ± 66.50	2912.46 ± 66.09	2909.26 ± 66.04	0.58
Protein, % of E	13.83 ± 0.24	14.22 ± 0.24	14.86 ± 0.24	0.01	14.10 ± 0.24	14.47 ± 0.24	14.34 ± 0.24	0.55
Carbohydrate,% of E	57.95 ± 0.63	57.87 ± 0.63	59.03 ± 0.63	0.35	57.21 ± 0.61	57.25 ± 0.60	60.39 ± 0.60	< 0.001
Fat, % of E	30.00 ± 0.62	29.23 ± 0.62	27.32 ± 0.62	0.01	30.53 ± 0.59	29.70 ± 0.59	26.33 ± 0.59	< 0.001
Cholesterol, mg	272.98 ± 12.18	290.58 ± 12.04	282.50 ± 12.04	0.59	285.82 ± 11.86	302.54 ± 11.77	257.88 ± 11.76	0.02
SFA, gr	27.44 ± 0.71	28.44 ± 0.70	26.17 ± 0.70	0.07	28.58 ± 0.66	28.99 ± 0.66	24.50 ± 0.66	< 0.001
MUFA, gr	28.99 ± 0.83	27.72 ± 0.82	25.95 ± 0.82	0.03	30.06 ± 0.79	28.21 ± 0.78	24.41 ± 0.78	< 0.001
PUFA, gr	29.51 ± 0.98	29.12 ± 0.97	26.83 ± 0.97	0.11	29.59 ± 0.97	28.92 ± 0.96	26.95 ± 0.96	0.13
Vitamin C, mg	180.89 ± 5.90	124.83 ± 5.83	95.86 ± 5.83	< 0.001	177.71 ± 5.75	133.37 ± 5.71	90.45 ± 5.70	< 0.001
Vitamin A, RAE	1525.04 ± 68.89	1011.76 ± 68.11	791.61 ± 68.09	< 0.001	1524.40 ± 66.51	1073.49 ± 66.02	730.50 ± 65.96	< 0.001
Thiamin, mg	2.56 ± 0.03	2.64 ± 0.03	2.73 ± 0.03	0.01	2.50 ± 0.03	2.63 ± 0.03	2.79 ± 0.03	< 0.001
Riboflavin, mg	2.45 ± 0.06	2.35 ± 0.06	2.10 ± 0.06	0.01	2.55 ± 0.06	2.41 ± 0.06	1.93 ± 0.06	< 0.001
Niacin, mg	26.13 ± 0.41	27.67 ± 0.40	28.87 ± 0.40	< 0.001	25.68 ± 0.37	27.41 ± 0.37	29.58 ± 0.37	< 0.001
Vitamin B6, mg	1.85 ± 0.04	1.57 ± 0.04	1.44 ± 0.04	< 0.001	1.87 ± 0.04	1.68 ± 0.04	1.31 ± 0.04	< 0.001
Vitamin E, mg	31.47 ± 1.41	31.59 ± 1.40	28.01 ± 1.40	0.12	30.37 ± 1.41	30.49 ± 1.40	30.20 ± 1.40	0.99
Folate, mcg	368.96 ± 11.59	305.05 ± 11.46	276.63 ± 11.45	< 0.001	381.34 ± 10.51	317.89 ± 10.43	251.58 ± 10.42	< 0.001
Vitamin B12, mcg	4.65 ± 0.18	4.61 ± 0.18	4.03 ± 0.18	0.02	4.98 ± 0.16	4.70 ± 0.16	3.62 ± 0.16	< 0.001
Magnesium, mg	323.79 ± 6.94	289.48 ± 6.86	251.79 ± 6.86	< 0.001	335.58 ± 5.74	296.15 ± 5.70	233.51 ± 5.69	< 0.001
Zinc, mg	10.85 ± 0.28	10.79 ± 0.27	10.25 ± 0.27	0.24	11.39 ± 0.25	11.08 ± 0.25	9.44 ± 0.25	< 0.001
Selenium, mcg	0.08 ± 0.01	0.09 ± 0.01	0.10 ± 0.01	0.01	0.08 ± 0.01	0.09 ± 0.01	0.09 ± 0.01	0.08
Total fiber, gr	23.11 ± 0.50	18.40 ± 0.50	16.87 ± 0.50	< 0.001	23.14 ± 0.48	18.85 ± 0.48	16.39 ± 0.48	< 0.001
Sodium, mg	3764.76 ± 140.53	3921.70 ± 138.93	4276.50 ± 138.88	0.03	3743.05 ± 139.80	4128.98 ± 138.77	4090.60 ± 138.65	0.10
Potassium, mg	4055.15 ± 89.91	3358.72 ± 88.88	2729.53 ± 88.85	< 0.001	4178.22 ± 72.91	3435.63 ± 72.37	2531.36 ± 72.31	< 0.001
`Calcium, mg	1416.07 ± 41.43	1381.51 ± 40.96	1215.82 ± 40.95	0.01	1483.59 ± 38.55	1384.78 ± 38.26	1146.02 ± 38.23	< 0.001
Phosphorus, mg	1514.80 ± 42.17	1504.23 ± 41.69	1400.54 ± 41.68	0.10	1617.77 ± 38.03	1532.18 ± 37.74	1271.14 ± 37.71	< 0.001

^1^Values are Mean ± SE. Energy intake, and macro-nutrients (protein, carbohydrate and fat) were adjusted for age and gender; all other values were adjusted for age, gender and energy intake. Abbreviations: NEAP: net endogenous acid production; PRAL: potential renal acid load; E: energy intake; SFA, Saturated fatty acids; MUFA, Monounsaturated fatty acids; PUFA, Polyunsaturated fatty acids.

^2^P value obtained from ANCOVA test.

**Figure 1:**
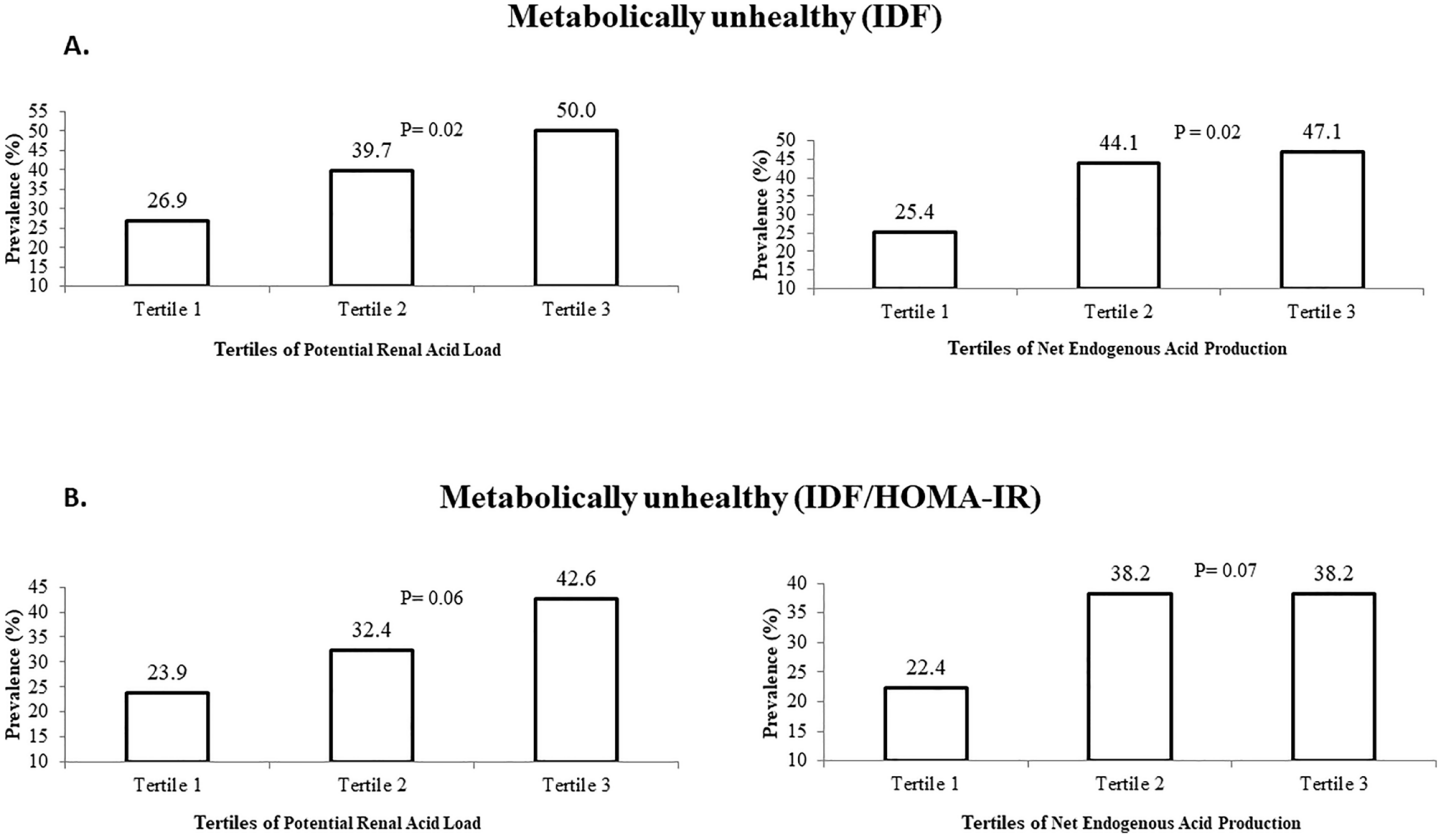
Prevalence of MUO across tertiles of PRAL and NEAP. (**A**) Based on IDF definition, (**B**) Based on IDF/HOMA-IR definition.

The prevalence of MUO (based on IDF and IDF/HOMA-IR criteria) across tertiles of PRAL and NEAP are reported in Fig. 1. Based on the IDF definition for metabolic health status, the prevalence of MUO in the highest tertile of PRAL, compared to the bottom tertile, was significantly higher (50.0 vs. 26.9%, P = 0.02). Also, the higher scores of NEAP were associated with more prevalence of MUO (third tertile vs. first tertile of NEAP: 47.1 vs. 25.4%, P = 0.02). According to the second definition of MUO (IDF/HOMA-IR criteria), the prevalence of MUO in the top tertile of PRAL and NEAP vs. the bottom tertile was marginally significant (42.6 vs. 23.9%, P = 0.06 and 38.2 vs. 22.4%, P = 0.07, respectively).

Crude and multivariate adjusted odds ratio and 95% CI for MUO phenotype across tertiles of PRAL and NEAP are shown in Table 3. In the crude model, adolescents in the top category of PRAL compared with the bottom level had 172% higher odds of MUO status, based on IDF criteria (OR 2.72; 95% CI 1.32–5.59). This association was significant after adjustment for age, sex, and energy intake (OR 2.42; 95% CI 1.13–5.15). But further adjustment for physical activity levels, SES, and BMI removed the significant association between PRAL categories and odds of MUO phenotype (fully-adjusted model: OR 1.55; 95% CI 0.65–3.66). The same findings were obtained for the association between NEAP tertiles with odds of MUO phenotype (crude model: OR 2.61; 95% CI 1.26–5.41; model 1: OR 2.52; 95% CI 1.17–5.41; and fully-adjusted model: OR 1.47; 95% CI 0.61–3.49). According to the IDF/HOMA-IR definition, the significant direct association between the highest category of PRAL and odds of MUO phenotype was observed only in the crude model (OR 2.37; 95% CI 1.13–4.96). This association was not significant in the crude model for NEAP. After adjustments for potential confounders, no significant relation was found between PRAL or NEAP and MUO phenotype (fully-adjusted model: OR 1.13; 95% CI 0.46–2.79; OR 1.21; 95% CI 0.48–3.07, respectively).

**Table 3:** Multivariate adjusted odds ratio (OR) and 95% confidence interval (CI) for MUO phenotype across tertiles of PRAL and NEAP ^1^.

	Tertiles of PRAL				Tertiles of NEAP			
	T1 (n = 67)	T2 (n = 68)	T3 (n = 68)	P trend^2^	T1 (n = 67)	T2 (n = 68)	T3 (n = 68)	P trend^2^
**MUO phenotype based on IDF criteria**								
Cases (n)	18	27	34		17	30	32	
Crude	1 (Ref.)	1.79 (0.86, 3.70)	2.72 (1.32, 5.59)	0.01	1 (Ref.)	2.32 (1.12, 4.81)	2.61 (1.26, 5.41)	0.01
Model 1	1 (Ref.)	1.71 (0.79, 3.71)	2.42 (1.13, 5.15)	0.02	1 (Ref.)	2.62 (1.20, 5.74)	2.52 (1.17, 5.41)	0.02
Model 2	1 (Ref.)	1.18 (0.49, 2.81)	1.55 (0.65, 3.65)	0.31	1 (Ref.)	1.97 (0.83, 4.67)	1.45 (0.61, 3.46)	0.43
Model 3	1 (Ref.)	1.13 (0.47, 2.71)	1.55 (0.65, 3.66)	0.30	1 (Ref.)	1.87 (0.78, 4.49)	1.47 (0.61, 3.49)	0.42
**MUO phenotype based on HOMA-IR criteria**								
Cases (n)	16	22	29		15	26	26	
Crude	1 (Ref.)	1.52 (0.71, 3.25)	2.37 (1.13, 4.96)	0.02	1 (Ref.)	2.14 (1.01, 4.56)	2.14 (1.01, 4.56)	0.05
Model 1	1 (Ref.)	1.34 (0.59, 3.03)	1.93 (0.88, 4.23)	0.09	1 (Ref.)	2.56 (1.11, 5.86)	2.20 (0.97, 4.95)	0.07
Model 2	1 (Ref.)	0.83 (0.32, 2.10)	1.16 (0.47, 2.83)	0.69	1 (Ref.)	1.84 (0.73, 4.64)	1.21 (0.48, 3.05)	0.78
Model 3	1 (Ref.)	0.76 (0.29, 1.96)	1.13 (0.46, 2.79)	0.70	1 (Ref.)	1.67 (0.65, 4.28)	1.21 (0.48, 3.07)	0.76

^1^All values are odds ratios and 95% confidence intervals. Model 1: Adjusted for age, sex, and energy intake. Model 2: Additionally, adjusted for physical activity and socioeconomic status (evaluated based on parental education level, parental job, number of family members, having car in the family, having computer/laptop, having personal room and having travel by using demographic questionnaire). Model 3: Additionally, adjusted for BMI.

^2^Obtained by the use of tertiles of PRAL or NEAP as an ordinal variable in the model.

Table 4 describes crude and multivariate adjusted odds ratio and 95% CI for MUO phenotype across tertiles of PRAL and NEAP, stratified by BMI categories. PRAL categories were not significantly associated with MUO status in overweight or in obese adolescents, based on both metabolic health status definitions. Among overweight adolescents, the highest category of NEAP, compared to the reference category, had nearly four times higher odds of MUO status, based on IDF criteria, in the crude model and model 1 (crude model: OR 4.04; 95% CI 1.35–12.06; model 1: OR 4.82; 95% CI 1.47–15.76). However, after adjustment for physical activity and SES, this association disappeared (fully-adjusted model: OR 2.03; 95% CI 0.47–8.75). When we examined these associations based on the IDF/HOMA-IR criteria, the same findings were found (crude model: OR 3.96; 95% CI 1.13–13.86; model 1: OR 4.96; 95% CI 1.23–19.89; and fully-adjusted model: OR 1.44; 95% CI 0.26–7.71).

**Table 4:** Multivariate adjusted odds ratio (OR) and 95% confidence interval (CI) for MUO phenotype across tertiles of PRAL and NEAP, stratified by BMI categories^1^.

	Tertiles of PRAL				Tertiles of NEAP			
	T1	T2	T3	P-trend^2^	T1	T2	T3	P-trend^2^
**MUO phenotype based on IDF criteria**								
**Overweight**								
(Participants/cases)	45/8	30/10	29/10		40/6	28/7	36/15	
Crude	1 (Ref.)	2.31 (0.78, 6.79)	2.43 (0.82, 7.18)	0.09	1 (Ref.)	1.88 (0.55, 6.38)	4.04 (1.35, 12.06)	0.01
Model 1	1 (Ref.)	3.22 (0.97, 10.60)	3.21 (0.97, 10.59)	0.05	1 (Ref.)	2.39 (0.63, 9.01)	4.82 (1.47, 15.76)	0.01
Model 2	1 (Ref.)	2.45 (0.59, 10.15)	1.86 (0.44, 7.75)	0.38	1 (Ref.)	1.15 (0.24, 5.39)	2.03 (0.47, 8.75)	0.31
**Obese**								
(Participants/cases)	22/10	38/17	39/24		27/11	40/23	32/17	
Crude	1 (Ref.)	0.97 (0.33, 2.79)	1.92 (0.66, 5.53)	0.17	1 (Ref.)	1.96 (0.73, 5.30)	1.64 (0.58, 4.64)	0.37
Model 1	1 (Ref.)	0.82 (0.26, 2.53)	1.51 (0.50, 4.56)	0.35	1 (Ref.)	2.20 (0.76, 6.34)	1.48 (0.49, 4.42)	0.51
Model 2	1 (Ref.)	0.54 (0.15, 1.95)	0.89 (0.24, 3.23)	0.98	1 (Ref.)	2.41 (0.74, 7.87)	1.11 (0.32, 3.84)	0.92
**MUO phenotype based on HOMA-IR criteria**								
**Overweight**								
(Participants/cases)	45/6	30/7	29/7		40/4	28/5	36/11	
Crude	1 (Ref.)	1.97 (0.59, 6.60)	2.06 (0.61, 6.93)	0.22	1 (Ref.)	1.95 (0.47, 8.05)	3.96 (1.13, 13.86)	0.02
Model 1	1 (Ref.)	2.67 (0.70, 10.20)	2.52 (0.66, 9.52)	0.17	1 (Ref.)	2.75 (0.57, 13.21)	4.96 (1.23, 19.89)	0.02
Model 2	1 (Ref.)	1.92 (0.38, 9.66)	1.13 (0.24, 5.34)	0.91	1 (Ref.)	1.06 (0.17, 6.58)	1.44 (0.26, 7.71)	0.62
**Obese**								
(Participants/cases)	22/10	38/15	39/22		27/11	40/21	32/15	
Crude	1 (Ref.)	0.78 (0.27, 2.26)	1.55 (0.54, 4.44)	0.30	1 (Ref.)	1.60 (0.59, 4.31)	1.28 (0.45, 3.61)	0.67
Model 1	1 (Ref.)	0.61 (0.19, 1.93)	1.15 (0.38, 3.50)	0.60	1 (Ref.)	1.81 (0.62, 5.23)	1.15 (0.38, 3.48)	0.85
Model 2	1 (Ref.)	0.38 (0.10, 1.46)	0.70 (0.19, 2.58)	0.80	1 (Ref.)	2.01 (0.61, 6.59)	0.87 (0.25, 3.07)	0.75

^1^All values are odds ratios and 95% confidence intervals. Model 1: Adjusted for age, sex, and energy intake. Model 2: Additionally, adjusted for physical activity and socioeconomic status (evaluated based on parental education level, parental job, number of family members, having car in the family, having computer/laptop, having personal room and having travel by using demographic questionnaire).

^2^Obtained by the use of tertiles of PRAL or NEAP as an ordinal variable in the model.

## Discussion

We found significant direct associations between PRAL and NEAP with odds of MUO status in Iranian adolescents, based on IDF criteria. However, these associations were dependant to covariates. The same finding was observed between PRAL and odds of MUO based on IDF/HOMA-IR definition, before adjustments for potential confounders. Stratified analysis indicated that greater NEAP categories were associated with higher odds for MUO among overweight adolescents, based on both definitions of metabolic health status. The present study is one of the first investigations on the association between DAL and metabolic health status in adolescents. The attainment of MHO may be a worthwhile first goal in the management of obesity and its complications, although MHO is an intermediate state before the development of MUO. Diet is one of the most important factors in determining obesity phenotype that should be considered to improve general health status^13^.

Our study showed possible beneficial relations between lower acid load of diet and metabolic health. Some prior studies have also investigated the association between DAL and obesity or metabolic syndrome. It is also worth noting that although some components of MHO/MUO and metabolic syndrome were the same, these are two different outcomes. Arisawa et al. have reported significant positive trends between NEAP scores with metabolic syndrome and some components of MetS in adults^35^. A systematic review and meta-analysis on observational studies additionally revealed that high DAL content was associated with higher obesity prevalence and serum TG concentrations^17^. A recent study demonstrated that the odds of obesity and abdominal adiposity significantly increased in tertile of DAL and also DAL could be an indicator of diet quality^36^. However, a previous cross-sectional study by Rahbarinejad et al. indicated no significant association between PRAL and NEAP and MetS in children before or after adjustment for potential confounders^24^. Also, another study found the same finding in adults, but higher scores of NEAP were associated with increased risk of impaired fasting blood glucose^37^. A national multi-center cross-sectional survey on 5326 Iranian students aged 6–18 years after evaluating anthropometric indices, showed a direct association between diet-induced acid load with neck circumference^23^. Furthermore, an inverse relationship was found between DAL indices and parental BMI^23^.

We observed that greater scores of PRAL and NEAP were related to higher FBG. Also, individuals with higher score of PRAL had higher TG. Some other investigations have also evaluated the relationship between DAL and cardiometabolic risk factors. A cross-sectional study on 371 Iranian adult women pointed out that women with higher NEAP scores had higher weight, WC, and TG concentrations. In case of PRAL, only a significant relation with TG was found^20^, which was similar to our finding. Lee et al. reported that diets with high acid-forming potential (high PRAL and NEAP scores) were positively associated with higher risk of IR during 7.3 years follow-up in a large population-based cohort study^38^. In another study on Japanese workers, high DAL was associated with IR, but not with FBG or HbA1c levels^21^. Another study by Haghighatdoost et al. demonstrated that PRAL and protein to potassium ratio were directly related to HbA1c among diabetic nephropathy patients^39^. Dehghan et al. revealed a potent negative relation between high DAL, particularly with PRAL scores, and cardiometabolic risk factors (including SBP, DBP, insulin, and odds of diabetes) in a systematic review and meta-analysis^40^. Differences in the findings of previous studies might be associated to the study populations, dietary intake assessment tools, sample size of the studies, applied methods to calculate dietary acid load, definition of dietary patterns, and various controlled confounders.

Several possible mechanisms might account for the relationship between DAL and MUO. Our study showed that adolescents in the top tertiles of PRAL and NEAP had greater intake of protein and carbohydrate, respectively. Animal food sources such as meat, fish, egg, chicken, cheese, and also cereals are rich in sulfur-containing amino acids, phosphorous and chloride. Consumption of these foods can contribute to higher acid production in the body; while high content of malate, citrate and glutamate in fruits and vegetables can contribute to base production. Therefore, animal-based foods, which are high in western diets, can be considered the most acid-producing diets and are associated with metabolic diseases^41^. These foods are pro-inflammatory and can increase inflammatory cytokines^42^, which are involved in lipid disorders and IR, particularly in obese subjects^43^. Higher DAL stimulates the production of glucocorticoids^44^. High cortisol levels induce lipase activity (lipoprotein lipase and hormone-sensitive lipase), which can lead to the increased efflux of fatty acids to the bloodstream and augment the production of very-low-density lipoproteins in the liver^45^. Furthermore, increased acidity of the body is related to the renal excretion of magnesium, calcium, and potassium^46^, although we did not measure serum concentration of these nutrients in our study. Increased BP and IR and obesity may result from these processes^47,48^. Also, diet-induced acidosis causes a decrease in adipokines (leptin and adiponectin), which can impair appetite suppression^49,50^. In addition, it has been demonstrated that high DAL reduces lean body mass and ultimately leads to increased body fat synthesis^51^.

The present study has some strength. First, the novelty of this study was the evaluation of DAL-metabolic health relation in a sample of adolescents from all socioeconomic districts of a large central city in Iran. Second, two different definitions of metabolic health status were used to determine MUO/MHO status. Third, several potential confounders were adjusted in the analysis. However, we acknowledge some limitations. The cross-sectional design of the study did not allow us to examine causality; so, conducting prospective studies is necessary. In addition, recall bias and other potential reporting biases may have affected the results. Also, the interview setting was used to collect dietary intakes data, which may be subject to social desirability biases. Despite controlling many potential confounders, residual confounders (such as the pattern of dietary habits, puberty, and sleep health) may affect the association between DAL scores and MUO. Finally, although BMI and WC were measured to define obesity and abdominal obesity, we could not measure body composition and fat distribution that could be involved in metabolic health status.

In conclusion, our findings indicated that diet-induced acid load might be associated with higher odds of MUO phenotype in overweight/obese adolescents. Thus, lower acidogenic food ingredients in the diets and high consumption of fruits and vegetables may improve the metabolic health status of adolescents. Further studies, particularly prospective nature, are needed to confirm these findings.

## Data Availability

The datasets generated and/or analysed during the current study are not publicly available as per the rules and regulations of the Isfahan University of Medical Science but are available upon reasonable request from the corresponding author.

## Abbreviations

DAL: Dietary acid load
PRAL: Potential renal acid load
NEAP: Net endogenous acid production
FFQ: Food frequency questionnaire
OR: Odds ratios
95% CI: 95% Confidence interval
BMI: Body mass index
MHO: Metabolically healthy obese
MUO: Metabolically unhealthy obese
IDF: International Diabetes Federation
HOMA-IR: Homeostasis Model Assessment Insulin Resistance
IR: Insulin resistance
PAQ-A: Physical Activity Questionnaire for Adolescents
SES: Socioeconomic status
SFA: Saturated fatty acids
MUFA: Mono unsaturated fatty acids
PUFA: Poly unsaturated fatty acids
WHO: World Health Organization
WC: Waist circumference
BP: Blood pressure
SBP: Systolic blood pressure
DBP: Diastolic blood pressure
TG: Triglycerides
HDL-c: High density lipoprotein cholesterol
FBG: Fasting blood glucose
ANOVA: One-way analysis of variance
ANCOVA: Analysis of covariance
SPSS: Statistical package for the social sciences
SD: Standard deviation
SE: Standard error
GBD: Global burden of disease
CVD: Cardiovascular disease
DASH: Dietary approaches to stop hypertension
NC: Neck circumference
